# Changes in oncogenic protein levels in peri-implant oral malignancy: a case report

**DOI:** 10.1186/s40902-019-0235-z

**Published:** 2019-11-08

**Authors:** Mi Hyun Seo, Hoon Myoung, Jong Ho Lee, Soung Min Kim, Suk Keun Lee

**Affiliations:** 1 Department of Oral and Maxillofacial Surgery, Dental Research Institute, School of Dentistry, Seoul National University, 101 Daehak-ro, Jongno-gu, Seoul, 110-768 South Korea; 2 Department of Oral Pathology, College of Dentistry, Gangneung-Wonju National University, 7, Jukheon-gil, Gangneung-si, Gangwon-do South Korea

**Keywords:** Oral squamous cell carcinoma (OSCC), Immunoprecipitation high-performance liquid chromatography (IP-HPLC), Oncogenic protein, Peri-implant oral malignancy (PIOM)

## Abstract

**Background:**

Oral squamous cell carcinoma (OSCC) constitutes a group of tumors that exhibit heterogeneous biology, histopathology, and clinical behaviors.

**Case presentation:**

A 73-year-old male had a whitish leukoplakia-like lesion around inflamed peri-implant area (#42, #43, and #44), and this lesion had transformed to OSCC within 3 years. He underwent mass resection, selective neck dissection, and reconstructive surgery. To detect any carcinogenesis progression, we examined the removed tumor tissue as well as the patient’s preoperative and postoperative sera to identify causative oncogenic proteins using immunoprecipitation high-performance liquid chromatography (IP-HPLC).

**Conclusions:**

The protein expression levels of p53, E-cadherin, β-catenin, MMP-10, HER2, NRAS, Met, HER2, and ERb were significantly lower in the serum collected on postoperative day 10 than in the preoperative serum, and if these proteins are consistently not elevated in the serum 3 months after surgery compared with the preoperative serum, these proteins can be potential oncogenic proteins. However, we also found that the serum extracted 3 months after the operation had elevated levels of oncogenic proteins compared with that of the preoperative and 10-day postoperative serum indicating the possibility of tumor recurrence. At postoperative follow-up period, ipsilateral neck metastasis and second primary lesion were found and additional surgery was performed to the patient. IP-HPLC using the patient’s serum shows the possibility of oncogenic protein detection. However, follow-up IP-HPLC data is needed to find out patient-specific prognostic factors.

## Background

Squamous cell carcinoma (SCC) is the most serious malignant tumor that frequently invades adjacent orofacial structures and spreads to cervical lymph nodes. Clinical and pathological behaviors of oral squamous cell carcinoma (OSCC) are highly variable in terms of oral ulceration, bone destruction, infiltrative growth, and tumor metastasis [1–3]. Treatment modalities can be decided based on clinical staging, pathologic diagnosis, general status of patient, or anatomic region of tumor presence. Clinical staging can be determined during the initial work-up, including a clinical exam, radiological exam with dental panorama, ultrasonography, computed tomography (CT), magnetic resonance imaging (MRI), and positron emission tomography-computed tomography (PET-CT). One of the primary treatments for OSCC is radical excision with or without adjuvant chemoradiotherapy, which has proven to be effective for locoregional control. Nevertheless, biomarkers involved in tumor recurrence and prognosis have not been identified for OSCC [4, 5]. Clinically, detection of tumor markers in the serum is easy and non-invasive. Squamous cell carcinoma antigen (SCC-Ag), cytokeratin, are well-known tumor-related markers [6]. We assumed that protein markers produced by the tumor can be reduced by tumor ablative surgery. The serum of cancer patient was used for protein analysis.

Immunoprecipitation high-performance liquid chromatography (IP-HPLC) is a type of protein detection method that is based on real antigen-antibody reaction in a phosphate-buffered saline (PBS) solution, followed by purification using protein A/G-conjugated agarose beads. Although its procedures are simple and easy to apply to most biological samples, IP-HPLC can yield a minimum error range by using micro-beads instead of small wells to mimic the enzyme-linked immunosorbent assay (ELISA) [7, 8]. The patient’s serum was collected before his operation, 10 days after the operation, and 3 months after the operation. Each sample was analyzed via IP-HPLC, which has been improved in terms of data accuracy and statistical analysis.

## Case presentation

A 73-year-old male patient was referred from his general dentist for further evaluation of whitish lesion on the attached gingiva and associated peri-implantitis. A panoramic view shows generalized alveolar bone loss and calculi deposition in the peri-implant region (#42, #43, #44), and the right mandibular anterior and premolar area showed peri-implant crestal bone loss. Laboratory findings were within normal limits. Other several oncogenic protein elevation situations, including tobacco and/or alcohol use, no nutritional deficiencies, no findings of ionizing radiation exposure, no immunodeficiency or immunosuppressant, and other removal prosthesis irritations, were all excluded. A whitish lesion was excised, and the specimen was sent to an oral pathologist. The contaminated implant surface was treated with a laser. The pathologic diagnosis was confirmed as oral candidiasis. The patient underwent laser treatment three times to treat the peri-implantitis lesion. One year later, his implant of #42, #43, and #44 area were removed due to peri-implantitis at the local clinic. From the referral letter of local clinic, the implant system was internal frictional connection type having a SLA surface, which was installed for more than 10 years.

**Fig. 1:**
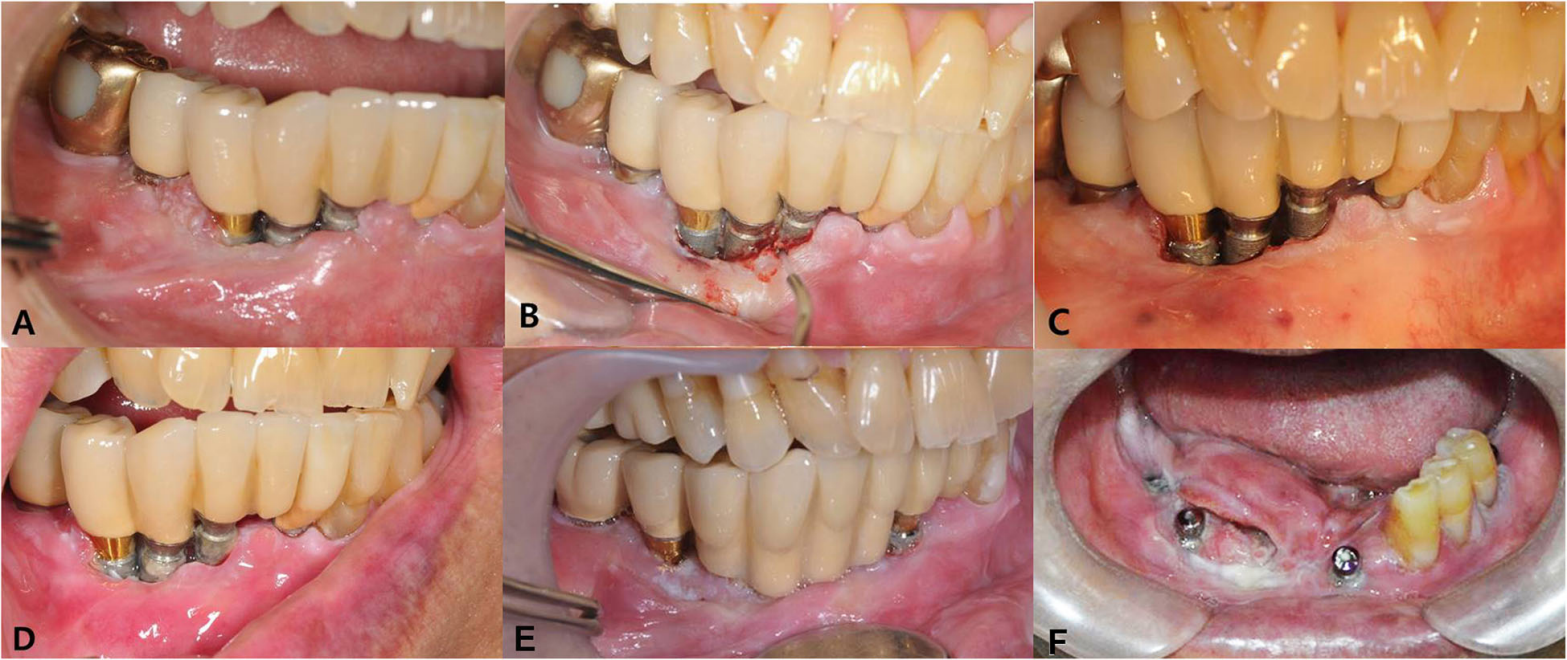
Clinical photos of the patient. **a** Initial visit; **b** peri-implantitis treatment with laser; **c** after 3-times laser treatment; **d** 5 months after initial visit; **e** 3 years after initial visit, the lesion was confirmed OSCC by incisional biopsy; **f** preoperative view show bulging tumor mass to lingual side

About 3 years later after the laser operation, a bulging mass was identified on the lingual side of #43 and #44 area. An incisional biopsy was performed and was diagnosed as a SCC (Fig. 1). Further work-up was performed including lab, chest X-ray, ECG, MRI, contrast CT, PET-CT, and neck sonography. The patient was diagnosed with cT4aN2cM0 stage IVA according to the TNM staging system proposed by the American Joint Committee on Cancer (AJCC, 2018). He was immediately scheduled for an operation that included selective neck dissection, mass resection with marginal mandibulectomy, and reconstruction with a radial forearm free flap. The final pathologic report was well-differentiated squamous cell carcinoma, with a 1.5 × 2.0-cm size of tumor, no metastasis to any of the 27 regional lymph nodes, and clear surgical resection margin. Vascular and perineural invasions were not observed; thus, he was diagnosed as pT1N0M0, stage I. Especially, rather than on the interface between the implant and the bone, tumor cells occurred on the surface of the mucosal soft tissue first and penetrated deeply along the implant threads. Neither adjuvant radiotherapy nor chemotherapy was not administered to the patient.

**Fig. 2:**
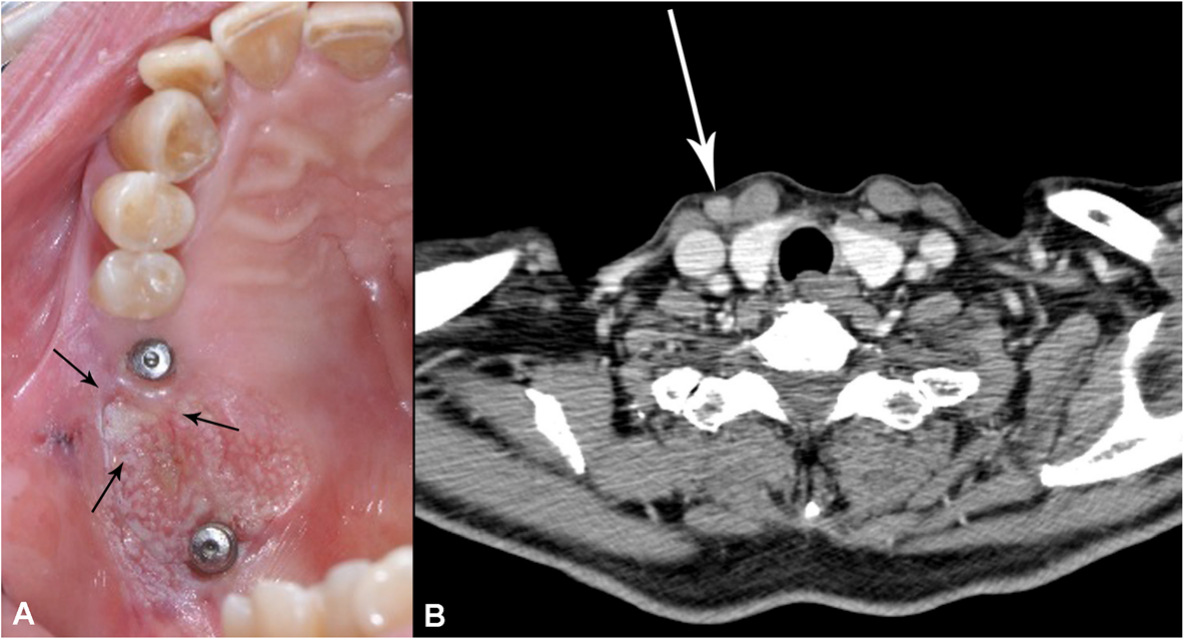
Clinical signs showed the recurrence of tumor. **a** Newly developed maxillary peri-implant oral malignancy lesion, **b** CT findings suspected of metastatic lymph node

A metastatic lymph node was found at the right ipsilateral level IV on enhanced CT taken 4 months postoperatively. Selective neck dissection, including right level IV, was performed, and a newly developed suspicious for malignant lesion was found on the right maxilla. The patient’s maxilla lesion was confirmed for SCC by incisional biopsy; therefore, he underwent an additional surgery 13 days after second selective neck dissection (Fig. 2). The final pathologic report on the maxillary lesion was well-differentiated SCC with a 2.0 × 1.4-cm size of tumor; depth of invasion was 0.7 cm. Involvement of underlying bone was present. Surgical resection margins were clear.

The surgically removed specimens were fixed in 10% neutral buffered formalin, processed following a routine protocol, and serial micro-sections with different antisera were also prepared for immunohistochemical staining. All data files of the patient were selected from the files of the Department of Oral and Maxillofacial Surgery, Seoul National University Dental Hospital under the approval of the institutional review board of Seoul National University (S-D20170026).

### IP-HPLC analysis for the protein extract obtained from the serum of patients

The patient’s blood was collected preoperatively, 10 days postoperatively, and 3 months postoperatively. After precipitation at room temperature, the samples were centrifuged at 4000 rpm for 20 min. Only the supernants were collected and mixed with lysis buffer and used for IP-HPLC. We applied 100 μg of each protein extract to the immunoprecipitation procedure using a protein A/G agarose column (Amicogen Co., Korea). The protein A/G agarose columns were separately pre-incubated with 1 μg of each of the 20 different antisera, including p53, muc1, muc4, TGF-β1, survivin, Wnt1, E-cadherin, β-catenin, matrix metalloproteinase (MMP)-3, MMP-10, TNFα, HER1, HER2, PAI-1, NRAS, KRAS, CEA, Met, FASL, and ERb. Briefly, the protein samples were mixed with 5 ml of binding buffer (150 mM NaCl, 10 mM Tris pH 7.4, 1 mM EDTA, 1 mM EGTA, 0.2 mM sodium vanadate, 0.2 mM PMSF, and 0.5% NP-40) and incubated in the protein A/G agarose columns at 10 °C for 1 h. The columns were placed on a rotating stirrer during the incubation. After washing each column with a sufficient amount of PBS solution (pH 7.3, 137 mM NaCl, 2.7 mM KCl, 43 mM Na_2_HPO_4_-7H_2_O, and 1.4 mM KH_2_PO_4_), the target protein was eluted with 150 μl of IgG elution buffer (Pierce Co., USA). The immunoprecipitated proteins were analyzed by HPLC (1100 series®, Agilent, USA) using a reverse-phase column and micro-analytical detector system (SG Hightech Co., Korea), operated with a 0.15-M NaCl and 20% acetonitrile solution at 0.4 mL/min for 30 min, and analyzed via UV spectroscopy at 280 nm. In the IP-HPLC results, the sample protein peak areas obtained from the HPLC analysis in the negative control were used to eliminate the antibody peak area (mAU*s) [7–9]. To compare preoperative and postoperative serum protein, the protein peak area values were proportionally normalized by the α-tubulin value and plotted as a bar.

### IP-HPLC analysis of extracted tumor protein

Protein extracts were prepared from tumor tissue, 100 μg each protein extract to the immunoprecipitation procedure using a protein A/G agarose column. The protein A/G agarose columns were individually pre-incubated with 1 μg of each of the 9 different antisera, including TNFα, NRAS, HER2, Met, E-cadherin, p53, survivin, TGF-β1, and NFκB. Briefly, the protein samples were mixed with 5 ml of binding buffer (150 mM NaCl, 10 mM Tris pH 7.4, 1 mM EDTA, 1 mM EGTA, 0.2 mM sodium vanadate, 0.2 mM PMSF, and 0.5% NP-40) and incubated in the protein A/G agarose columns at 10 °C for 1 h. To compare the normal gingiva and SCC tissue protein, the protein peak area values were proportionally normalized by the α-tubulin value and plotted as a bar.

### Laboratory analysis

Routine laboratory results were collected, including complete blood cell counts (CBC) with differential count, C-reactive protein (CRP) levels, and erythrocyte sedimentation rate (ESR). A modest elevation in the plasma CRP in the range observed in apparently healthy individuals is a strong predictor of future vascular events [10]. Chen et al. [11] reported the presence of elevated preoperative serum CRP level (> 5.0 mg/L) is an independent prognostic indicator of oral cancer. The results from the blood sample tests were compared from the first visit, preoperatively, postoperatively, and at the time of recurrence.

### IP-HPLC analysis from the serum of patients

**Fig. 3:**
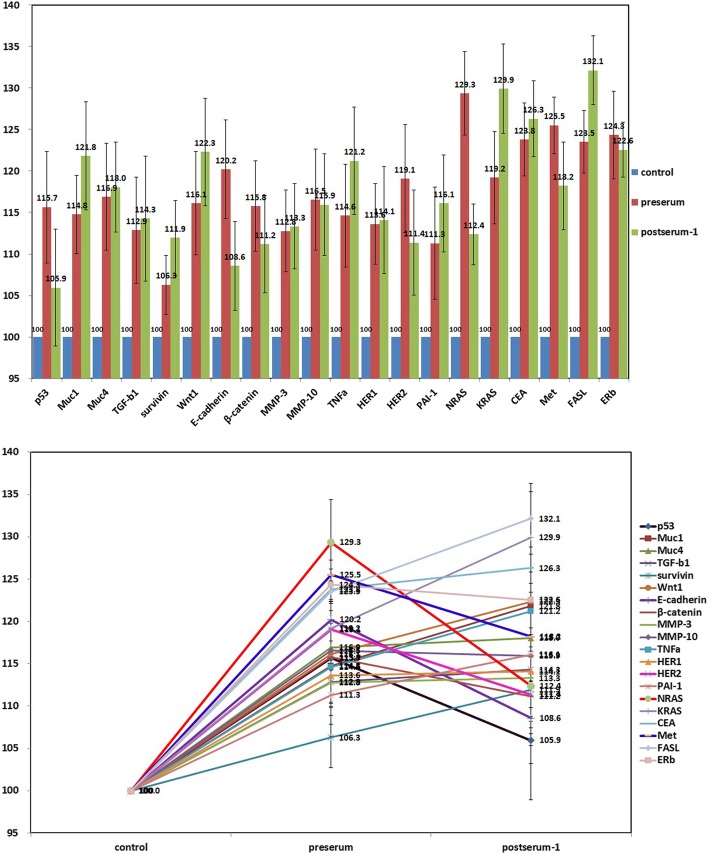
An upper bar graph and a lower line graph comparing oncogenic protein expression profiles between preoperative and postoperative day 10 serum

**Fig. 4:**
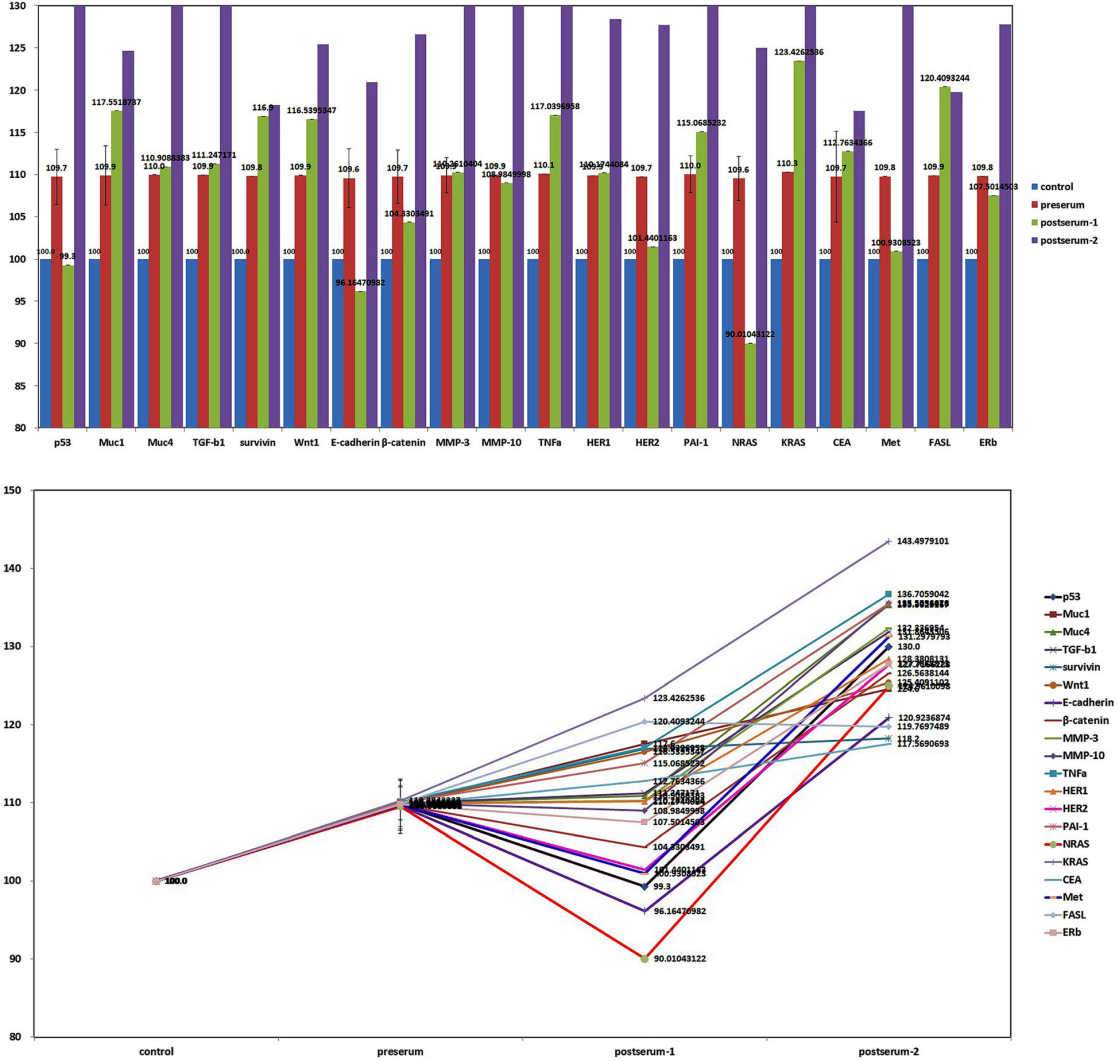
An upper bar graph and a lower line graph comparing oncogenic protein expression profiles between preoperative, postoperative 10 days, and postoperative 3 months after first ablation surgery

The IP-HPLC analyses revealed that p53, E-cadherin, β-catenin, MMP-10, HER2, NRAS, Met, and ERb had decreased at postoperative day 10. Other protein markers related to oncogenic signaling were increased at postoperative day 10. This suggests that oncogenic proteins, such as p53, E-cadherin, β-catenin, MMP-10, HER2, NRAS, Met, and ERb were released from the primary tumor; therefore, serum levels of these oncogenic proteins decreased after tumor-ablative surgery (Fig. 3). In 3 months postoperatively using the patient’s serum, all oncogenic protein markers were elevated and tumor recurrence or metastasis was suspected (Fig. 4).

### IP-HPLC analysis from extracted tumor protein

**Fig. 5:**
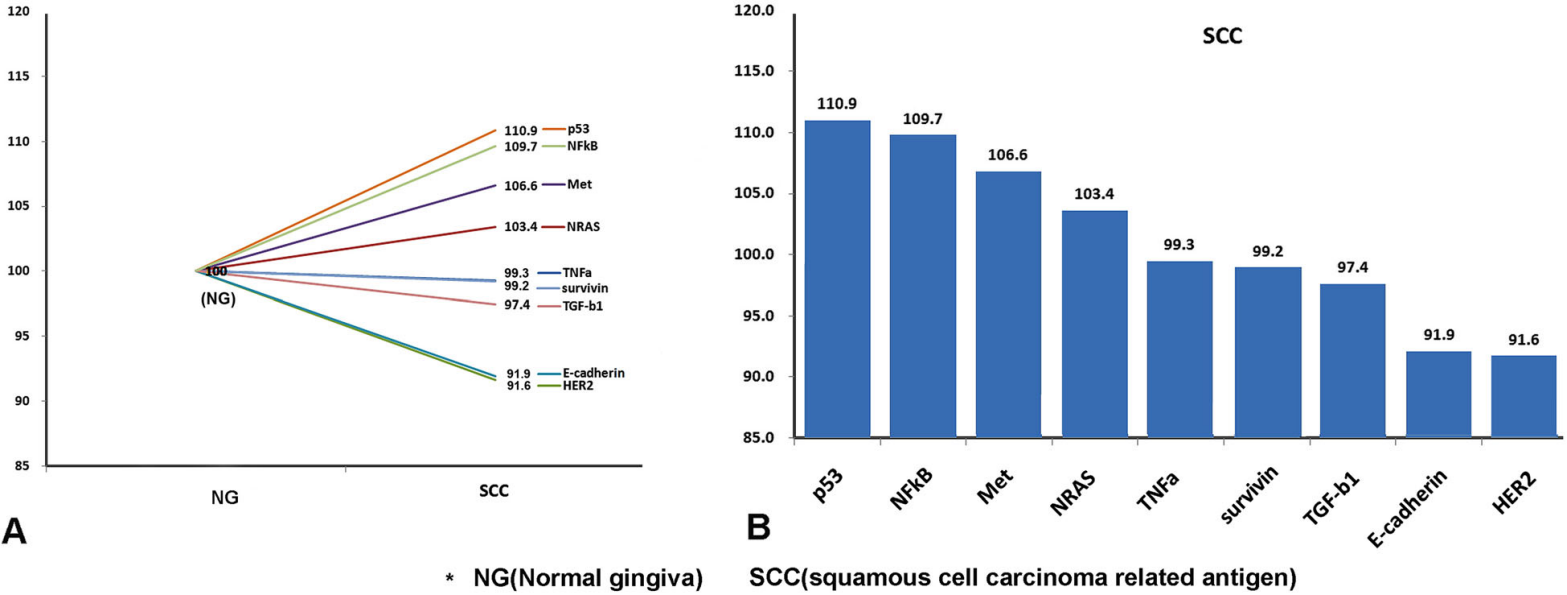
A line graph (**a**) and a bar graph (**b**) comparing oncogenic protein expression profiles between normal gingiva and SCC tissue from the first ablation surgical specimen

IP-HPLC analyses revealed that NRAS, Met, p53, and NFκB were overexpressed in the SCC tissue in the comparison of oncogenic protein levels between normal gingiva and SCC tissue (Fig. 5).

### Laboratory findings

Inflammatory markers such as white blood cell count (WBC), absolute neutrophil count (ANC), ESR, and CRP were elevated immediately postoperatively. CRP was not conducted preoperatively, and so, that data cannot be compared to other time points; however, CRP levels had not changed significantly before and after the second operation (right level IV selective neck dissection, Additional file 1: Figure S1). Notably, ESR counts were elevated in the preoperative period, as determined when a malignant lesion was confirmed by incisional biopsy and compared with a sample taking at the first visit. That indicates that some inflammatory reactions can affect the potential of malignant transformation.

Perioperative changes in the RBCs, hemoglobin, hematocrit showed that the levels were decreased during the post-surgery, but tended to recover over time (Additional file 2: Figure S2). The proportion of segmented neutrophils increased immediately after surgery, which are characteristic cells of acute inflammatory reactions, because they moved to surgical wound immediately after trauma in several minutes. The graphed curves of lymphocyte, monocyte, eosinophil, and basophil levels showed opposite trends from the segmented neutrophil levels (Additional file 3: Figure S3).

## Discussion

The peri-implant leukoplakia lesion and peri-implantitis transformed to SCC over several years in this patient; oral cancers may be preceded by potentially malignant disorders, recognizable mucosal diseases such as localized leukoplakia or erythroplakia, or other wide-spread conditions, all of which harbor a considerably increased risk for SCC [12].

Peri-implantitis is a common long-term complication of dental implant. Clinical characteristics of peri-implantitis include swelling, erythema, and often suppuration. Marginal bone loss and pocket formation are typical. The etiology is multifactorial and not fully understood, with bacterial factors, surgical factors, implant surface characteristics, prosthetic design, and genetic predisposition suggested as contributing factors. A review of several small case series included analyses of biopsy material from peri-implantitis, reported hyperplasia, and ulceration of pocket epithelium and presence of a mixed population of inflammatory cells [13]. A study of 117 biopsies from peri-implantitis cases reported that nearly 50% of cases did not exhibit simple inflammatory changes. Instead, other potentially aggressive lesions, such as pyogenic granuloma, giant cell granuloma, or *Actinomyces*-related inflammation were diagnosed [14]. A literature review of retrospective analyses of cases from 2000 to 2016 revealed 47 reported cases of oral malignancy involving dental implants. Peri-implant malignancies were primary tumors in 29 cases (61.7%), recurrent or second primary tumors in 11 cases (23.4%), and metastasis from distant tumors in 4 cases (8.5%). Recognized risk factors for oral cancer included potentially malignant conditions (erythroplakia, leukoplakia, oral lichen planus, or proliferative verrucous leukoplakia) in 21 cases (44.7%). There is very little evidence that links dental implant and cancer. Regarding orthopedic stainless-steel plates, an annual prevalence increase of 0.12% per year for osteosarcoma has been reported and is suggested to be a result of corrosion caused by surface imperfections and chronic inflammation [15]. No such data exist regarding titanium dental implants. Only a single in vitro experiment found that exposure to titanium particles yielded a dose-responsive induction of chromosomal instability in human fibroblasts, similar to that induced by heavy metal and low-dose radiation exposure. There are conflicting results in the literature, with no clear evidence that corrosion material (as opposed to titanium oxide nanoparticles) can play a role in carcinogenesis [16, 17]. However, peri-implant malignancy is not as rare as currently reported and may be responsible for 1.5% of oral malignancy cases [18].

Numerous candidate biomarkers have been evaluated in blood, tumor, and saliva specimens, which yields hopes elucidating disease pathways and improved prognosis prediction. Several classes of molecules of particular interest have been evaluated, including MMPs, interleukins, proangiogenic and antiangiogenic factors, growth factors, and tumor factors [19]. In this study, we evaluated the oncogenic proteins using preoperative and postoperative serum and tumor tissue via IP-HPLC. Serum samples were collected preoperatively, 10 days postoperatively (before recurrence detection), and 3 months postoperatively (between the first tumor ablation surgery and the second dissection of metastatic lymph node). p53, E-cadherin, β-catenin, MMP-10, HER2, NRAS, Met, and ERb were lower in the serum collected on the postoperative 10 days. NRAS, Met, p53, and NFκB were overexpressed in the tumor compared with the normal gingiva. Therefore, we considered oncogenic proteins including p53, E-cadherin, β-catenin, MMP-10, HER2, NRAS, Met, and ERb as prognostic markers in this tumor. p53 tumor suppressor gene is essential in regulating cell cycle progression, differentiation, DNA repair, and apoptosis. p53 level in saliva was shown to be significantly higher in patients with OSCC [20]. Cadherin is a superfamily of calcium-dependent transmembrane proteins that act as adhesion molecules with roles in a variety of processes including development, morphogenesis, organogenesis, and carcinogenesis. The cadherin switch is probably necessary for increased mobility but not for morphological changes that occur in epithelial-mesenchymal transition (EMT) [21]. However, controversy exists, some studies showing that a simple loss of E-cadherin is responsible for OSCC progression [22].

In Wnt signaling pathways, the β-catenin pathway is one of the major oncogenic pathways; deregulation of the β-catenin pathway and consequent overactivity of β-catenin may contribute to oral SCC progression. More recently, it has been shown that the activation of the β-catenin pathway is correlated with EMT and invasiveness of cancer cells [23]. Epidermal growth factor family of transmembrane tyrosine kinase receptors includes homodimers or heterodimers of four receptors: EGFR, HER2, HER3, and HER4. The role of HER2 in OSCC has failed to come up with definitive involvement [24]; however, in this study, the serum level of HER2 was decreased at postoperative day 10, showing downregulation in the tumor IP-HPLC. MMPs are a 24-member family of extracellular proteins subdivided into different groups based on structural characteristics and substrate specificity and include the following: collagenases, gelatinases, stromelysins, and membrane-type MMPs.

Cancer cells degrade the extracellular matrix facilitating migration and metastasis. OSCC progression is likely to be maintained by the stromelysins as tumor margins show expression of MMP-3, whereas MMP-10 and MMP-11 are linked to tumor differentiation and local invasion [25]. The Met tyrosine kinase is a heterodimeric cell-surface receptor consisting of a disulphide-linked 145-kDa transmembrane β-chain and an extracellular 50-kDa α-chain. Its only known agonistic ligand is hepatocyte growth factor (HGF). The cellular effect induced by HGF via Met includes enhanced proliferation, stimulation of cell movement and migration, and a complex regulation of cell survival and pro-apoptotic effects, depending on the biological context [26]. In OSCC, Met expression has been associated with cisplatin resistance and a strong metastatic ability in vivo, as well as a poor patient prognosis [27]. Ras gene family mutations are frequently associated with the development of cancer with each tumor type showing a predisposition to one of isoforms, e.g., HRAS, KRAS, and NRAS. Activated RAS facilitates several signaling cascades including PI3K/AKT and MAPK. Activated AKT encourages cell survival through inhibition of apoptosis and activation of NFκB [24, 27]. NFκB is a nuclear transcription factor that participates in cellular functions such as proliferation, survival, and apoptotic processes and also associated with inflammation, immunoregulation, and cancer. Some effectors of activated NFκB include chemokines and cytokines that sustain growth and confer advantages allowing tumor growth in the microenvironment through stimulation of inflammatory cell infiltration and angiogenesis. NFκB is also responsible for the enhanced expression of MMP degradative enzymes in many tumors, which may be linked to the ability of tumors to metastasize through the breakdown of supporting structures [28].

Overexpression of NRAS, Met, p53, and NFκB in tumor IP-HPLC revealed the potential of proliferation and metastasis. However, all of the oncogenic proteins evaluated in this study were increased in the serum at the 3-month postoperative period. Further IP-HPLC analysis using the patient’s serum is required for identification of prognostic markers. The IP-HPLC results from the serum samples allowed for early detection in tumor recurrence in this patient, and enhanced CT images, which were taken 4 months postoperatively, revealed a metastatic lymph node at ipsilateral level IV. Second primary lesion was also found on the maxillary gingiva around implant.

In this study, we showed that long-term inflammation, such as peri-implantitis, can contribute to premalignant mucosa disease which can subsequently change to malignancy. There was inevitable limitation in the way that we conduct simple comparison of preoperative and the 10-day postoperative sera of the patient to identify serum-based prognostic markers. IP-HPLC data from a longer postoperative follow-up period are needed to better understand the overall tendency of tumor behavior. Therefore, clinicians have to closely follow-up with cases of premalignant lesion, especially in the peri-implantitis cases.

## Conclusions

The protein expression levels of p53, E-cadherin, β-catenin, MMP-10, HER2, NRAS, Met, HER2, and ERb were significantly lower in the serum collected on postoperative day 10 than in the preoperative serum. At postoperative follow-up period, IP-HPLC using patient’s serum showed the possibility of oncogenic protein profiles detection. Follow-up IP-HPLC data is needed to find out patient-specific prognostic markers.

## Supplementary information

**Additional file 1: Figure S1.** Changes in the serum markers which can be used for evaluation of degree of inflammation showing WBC (A), ANC (B), ESR (C), and hs-CRP (D). IP-HPLC 1 indicated the timing of the serum collected for IP-HPLC analysis at postoperative 10 days. IP-HPLC 2 indicated the timing of the serum collected for IP-HPLC analysis at postoperative 3 months. The result of IP-HPLC 2 described the recurrence of tumor, and after enhanced CT taking, the selective neck dissection was performed to remove of metastatic lymph node.

**Additional file 2: Figure S2.** Changes of component of complete blood cell counts showing RBC (A), Hb (B), Hct (C), and PLT (D).

**Additional file 3: Figure S3.** Changes of differential count of CBC showing segmental neutrophil (A), lymphocyte (B), monocyte (C), eosinophil (D), and basophil (E).

## Data Availability

Data sharing is not applicable to this article as no data sets were generated or analyzed during the current study.

## Abbreviations

ANC: Absolute neutrophil count
CBC: Complete blood cell counts
CRP: C-reactive protein
CT: Computed tomography
ELISA: Enzyme-linked immunosorbent assay
EMT: Epithelial-mesenchymal transition
ESR: Erythrocyte sedimentation rate
HGF: Hepatocyte growth factor
IP-HPLC: Immunoprecipitation high-performance liquid chromatography
MMPs: Matrix metalloproteinases
MRI: Magnetic resonance imaging
OSCC: Oral squamous cell carcinoma
PBS: Phosphate-buffered saline
PET-CT: Positron emission tomography-computed tomography
PIOM: Peri-implant oral malignancy
WBC: White blood cell count

## References

[1] Shibuya Y, Hasegawa T, Akashi M (2013). Oral squamous cell carcinoma with multiple neck metastases--cases with more than ten pathologically positive lymph nodes in the unilateral side. J Oral Maxillofac Surg.

[2] Suslu N, Hosal AS, Aslan T (2013). Carcinoma of the oral tongue: a case series analysis of prognostic factors and surgical outcomes. J Oral Maxillofac Surg.

[3] Grimm M (2012). Prognostic value of clinicopathological parameters and outcome in 484 patients with oral squamous cell carcinoma: microvascular invasion (V+) is an independent prognostic factor for OSCC. Clin Transl Oncol.

[4] Liao CT, Wang HM, Ng SH (2006). Good tumor control and survivals of squamous cell carcinoma of buccal mucosa treated with radical surgery with or without neck dissection in Taiwan. Oral Oncol.

[5] Chen IH, Liao CT, Wang HM (2014). Using SCC antigen and CRP levels as prognostic biomarkers in recurrent oral cavity squamous cell carcinoma. PLoS One.

[6] DE Paz D, Young CK, Chien HT (2019). Prognostic roles of SCC antigen, CRP and CYFRA 21-2 in oral cavity squamous cell carcinoma. Anticancer Res.

[7] Kim YS (2015). Protein expression changes induced by cisplatin in an oral cancer cell line as determined by immunoprecipitation-based high performance liquid chromatography. Kor J Oral Maxillofac Pathol.

[8] Kim YS, Lee SK (2015). IP-HPLC analysis of human salivary protein complexes. Kor J Oral Maxillofac Pathol.

[9] Kim SM, Jeong D, Kim MK (2017). Two different protein expression profiles of oral squamous cell carcinoma analyzed by immunoprecipitation high-performance liquid chromatography. World J Surg Oncol.

[10] Hashimoto H, Kitagawa K, Hougaku H (2011). C-reactive protein is an independent predictor of the rate of increase in early carotid atherosclerosis. Circulation.

[11] Chen HH, Chen IH, Liao CT (2011). Preoperative circulating C-reactive protein levels predict pathological aggressiveness in oral squamous cell carcinoma: a retrospective clinical study. Clin Otolaryngol.

[12] van der Waal I (2009). Potentially malignant disorders of the oral and oropharyngeal mucosa; terminology, classification and present concepts of management. Oral Oncol.

[13] Berglundh T, Zitzmann NU, Donati M (2011). Are peri-implantitis lesions different from periodontitis lesions?. J Clin Periodontol.

[14] Kaplan I, Hirshberg A, Shlomi B (2015). The importance of histopathological diagnosis in the management of lesions presenting as peri-implantitis. Clin Implant Dent Relat Res.

[15] Boudrieau RJ, McCarthy RJ, Sisson RD (2005). Sarcoma of the proximal portion of the tibia in a dog 5.5 years after tibial plateau leveling osteotomy. J Am Vet Med Assoc.

[16] Coen N, Kadhim MA, Wright EG (2003). Particulate debris from a titanium metal prosthesis induces genomic instability in primary human fibroblast cells. Br J Cancer.

[17] Matsumoto M, Filho HN, Ferrari R (2014). Genotoxicity of endosseous implants using two cellular lineages in vitro. J Oral Implantol.

[18] Kaplan I, Zeevi I, Rosenfeld E (2017). Clinicopathological evaluation of malignancy adjacent to dental implants. Oral Surg Oral Med Oral Pathol Oral Radiol.

[19] Lessing AAD, Joseph AM, Lindgren BR (2017). Association of oral cavity and oropharyngeal cancer biomarkers in surgical drain fluid with patient outcomes. JAMA Otolaryngol Head Neck Surg.

[20] Agha-Hosseini F, Mirzaii-Dizgah I, Miri-Zarandi NS (2015). Unstimulated salivary p53 in patients with oral lichen planus and squamous cell carcinoma. Acta Med Iran.

[21] Maeda M, Johnson KR, Wheelock MJ (2005). Cadherin switching: essential for behavioral but not morphological changes during and epithelium-to-mesenchyme transition. J Cell Sci.

[22] Ukpo DC, Thorstad WL, Zhang Q (2012). Lack of association of cadherin expression and histopathologic type, metastasis, or patient outcome in oropharyngeal squamous cell carcinoma: a tissue microarray study. Head Neck Pathol.

[23] Iwai S, Yonekawa A, Harada C (2010). Involvement of the Wnt-β-catenin pathway in invasion and migration of oral squamous carcinoma cells. Int J Oncol.

[24] Sinevici N, O’Sullivan J (2016). Oral cancer: deregulated molecular events and their use as biomarkers. Oral Oncol.

[25] Brusevold IJ, Søland TM, Khuu C (2010). Nuclear and cytoplasmic expression of Met in oral squamous cell carcinoma and in an organotypic oral cancer model. Eur J Oral Sci.

[26] Cho YA, Kim EK, Heo SJ (2016). Alteration status and prognostic value of MET in head and neck squamous cell carcinoma. J Cancer.

[27] Murugan AK, Munirajan AK, Tsuchida N (2012). Ras oncogenes in oral cancer: the past 20 years. Oral Oncol.

[28] Chen Z, Yan B, van Waves C (2008). The role of the NF-kappa B transcriptome and proteome as biomarkers in human head and neck squamous cell carcinomas. Biomark Med.

