# Supplementary figures and images for: Changes in oncogenic protein levels in peri-implant oral malignancy: a case report
Source: Maxillofac Plast Reconstr Surg. 2019 Nov 8;41(1):46. doi: 10.1186/s40902-019-0235-z (PMC6838285; doi:10.1186/s40902-019-0235-z)

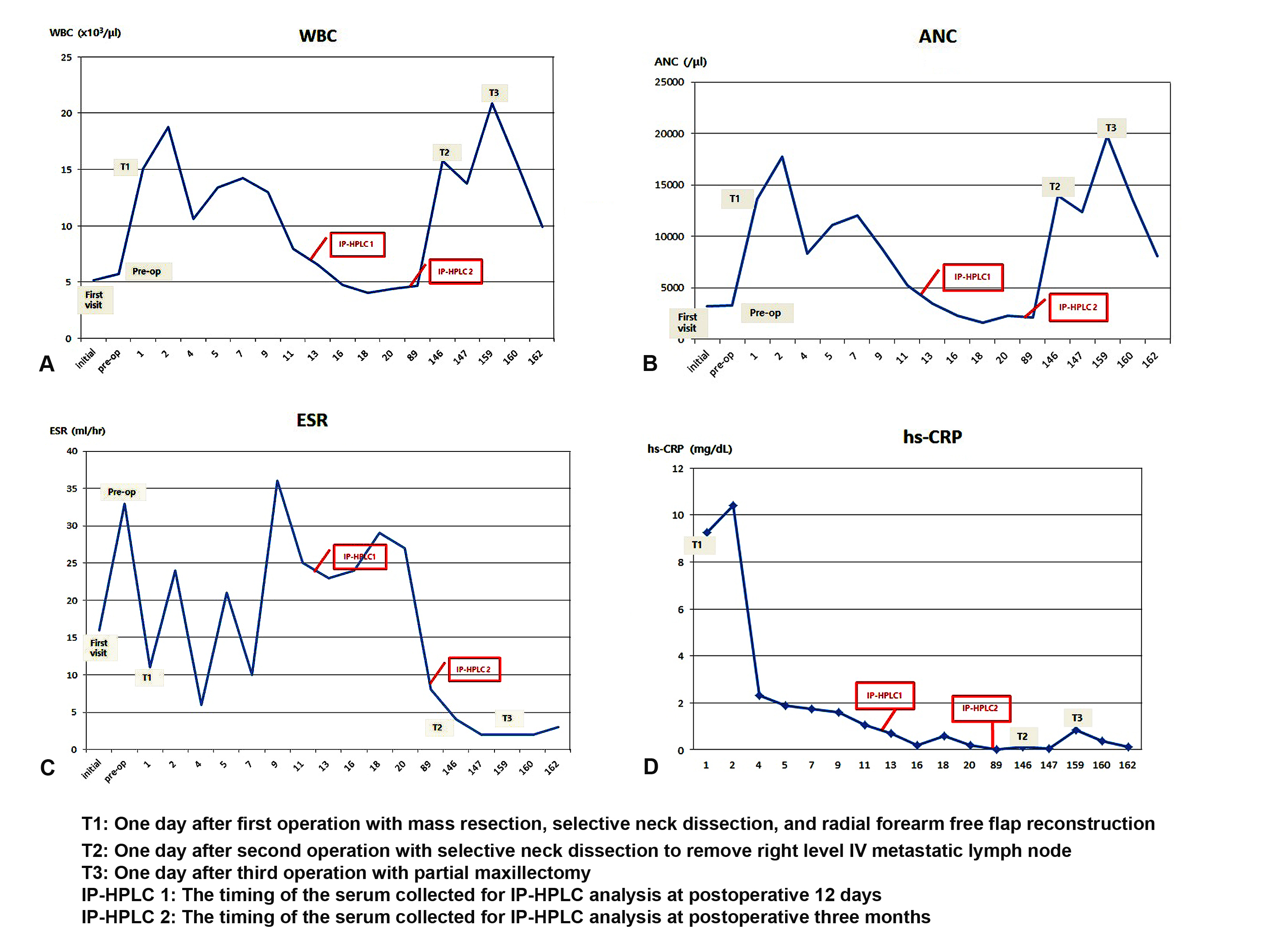

Supplement: Supplementary file 1 — Additional file 1: Figure S1. Changes in the serum markers which can be used for evaluation of degree of inflammation showing WBC (A), ANC (B), ESR (C), and hs-CRP (D). IP-HPLC 1 indicated the timing of the serum collected for IP-HPLC analysis at postoperative 10 days. IP-HPLC 2 indicated the timing of the serum collected for IP-HPLC analysis at postoperative 3 months. The result of IP-HPLC 2 described the recurrence of tumor, and after enhanced CT taking, the selective neck dissection was performed to remove of metastatic lymph node. [file 40902_2019_235_MOESM1_ESM.jpg]

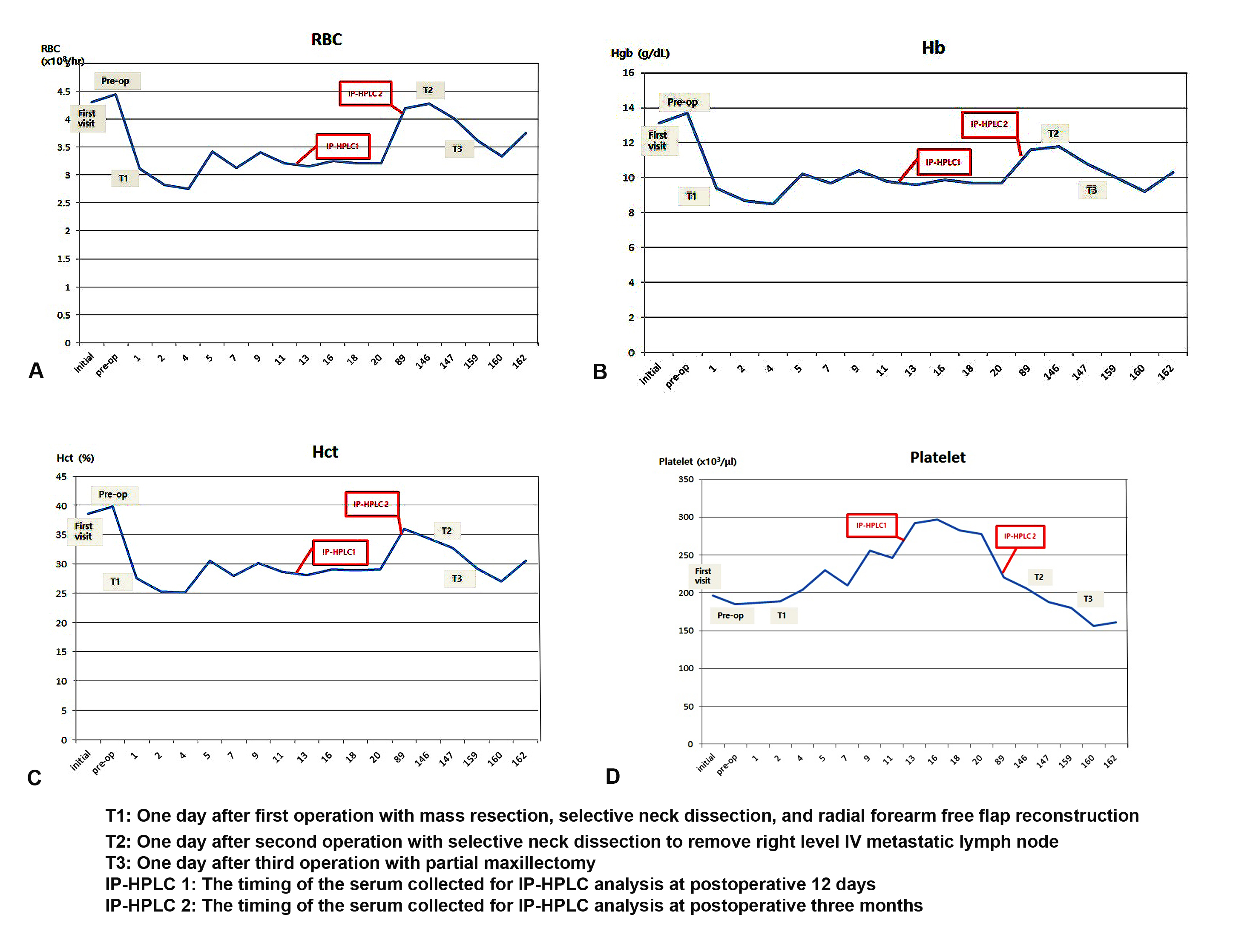

Supplement: Supplementary file 2 — Additional file 2: Figure S2. Changes of component of complete blood cell counts showing RBC (A), Hb (B), Hct (C), and PLT (D). [file 40902_2019_235_MOESM2_ESM.jpg]

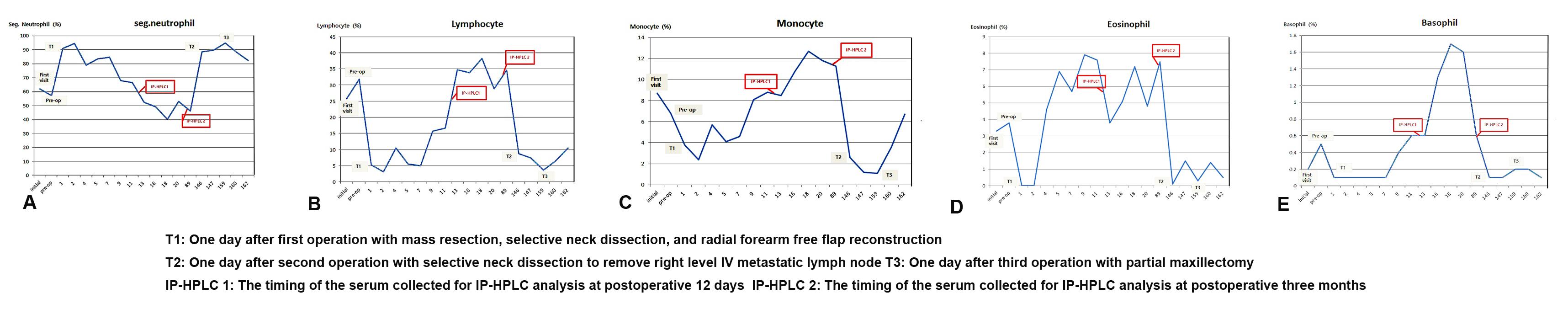

Supplement: Supplementary file 3 — Additional file 3: Figure S3. Changes of differential count of CBC showing segmental neutrophil (A), lymphocyte (B), monocyte (C), eosinophil (D), and basophil (E). [file 40902_2019_235_MOESM3_ESM.jpg]
